# Acute-on-Chronic Liver Failure: From Basic Research to Clinical Applications

**DOI:** 10.1155/2018/5029789

**Published:** 2018-10-08

**Authors:** En-Qiang Chen, Tetsuro Shimakami, Yu-Chen Fan, Paolo Angeli

**Affiliations:** ^1^Center of Infectious Diseases, West China Hospital of Sichuan University, Chengdu 610041, China; ^2^Department of Gastroenterology, Kanazawa University Graduate School of Health Medicine, Kanazawa, Japan; ^3^Department of Hepatology, Qilu Hospital of Shandong University, Jinan 250012, China; ^4^Department of Clinical and Experimental Medicine, University of Padova, Padova, Italy

Acute-on-chronic liver failure (ACLF) is a clinical syndrome of acute hepatic decompensation observed in patients with preexisting chronic liver disease (CLD) characterized by one or more extrahepatic organ failures with a significantly increased risk of death [1]. It is well known that the etiology of ACLF would be a precipitating event on a preexisting liver condition. However, no matter the etiology of preexisting chronic liver damage, ACLF patients are always with ascites, jaundice, portal hypertension with variceal bleeding, and/or hepatic encephalopathy [2]. Though the exact pathogenesis of ACLF is not fully elucidated in past decades, there is also significant progress in its basic and clinical research. There are still huge differences in the definitions of ACLF between the West and East [3]. As far as the precipitating events, in the West, acute alcohol injury and bacterial infections are the most common. In the East, this is true also for India and Korea, while in China reactivation of chronic hepatitis B is the most common.

ACLF can develop in patients with any underlying cirrhosis/CLD [1, 4]. According to our estimation, ACLF may be still a common severe liver disease in the coming decade. How to prevent the occurrence and development of ACLF and how to effectively treat and predict the prognosis of ACLF patients are still embarrassing problems that we have to face in real-life clinical practice. Nowadays, besides the prevention of precipitating factors leading to acute hepatic decompensation, the effective management of ACLF should also include active supportive care and earliest initiation of specific therapies against cytokine storm [5]. For ACLF patients with particularly serious condition, artificial liver support system and possible liver transplantation should be also considered.

This special issue encompasses basic and clinical studies as well as review article focusing on ACLF. It includes 6 research articles, 1 clinical study, and 1 review article describing the advance of the pathogenesis and clinical management of ACLF, and all of them are summarized as follows.

According to the review article titled “Systemic Inflammation and Acute-on-Chronic Liver Failure: Too Much, Not Enough,” dysbalanced immune function is central to ACLF's pathogenesis and outcome with an initial excessive systemic inflammatory response that drives organ failure and mortality. And the immunoexhaustion/immunoparalysis in later course prevails predisposing the patient to secondary infectious events and reescalation in end-organ dysfunction and mortality. In this review, W. Laleman et al. also mention that the management of ACLF patients is still poorly defined at present. However, with understanding of the pathophysiology of ACLF gradually deepened, potential therapeutic targets emerge that warrant further study such as restoring or substituting albumin via plasma exchange or via albumin dialysis and evaluating usefulness of TLR4 antagonists, modulators of gut dysbiosis, and FXR-agonists.

The research article titled “Management Strategies and Outcomes for Hyponatremia in Cirrhosis in the Hyponatremia Registry” aims to assess the treatment practices and effectiveness in cirrhotic patients with hyponatremia (HN) in the HN Registry. It is well-known that dilutional HN is a frequent consequence of severe portal hypertension in patients with cirrhosis and ACLF. In this research study, S. Sigal et al. have included 595 cirrhotic patients with HN and compared the characteristics, treatments, and outcomes between patients with HN at admission and during hospitalization. They found that patients with HN at admission have a lower [Na] and shorter length of stay (LOS) than those who develop HN; and the treatment approaches for HN are variable and frequently ineffective. However, success is greatest with hypertonic saline and tolvaptan; and relapse of HN is associated with increased LOS. The findings from the study have given us a deeper understanding of the effectiveness of treatment strategies and impact on LOS for hospitalized cirrhotic patients with HN.

The research article titled “The Correlation between miR-122 and Lipoprotein Lipase Expression in Chronic Hepatitis C Patients” aims to analyze the relationship between viral load, lipid profile, IFN*γ*, and the expression of miR-122 and LPL in the liver and PBMCs. As we know, miR-122 is the foremost liver-related micro-RNA [6], and it is reported that chronic hepatitis C (CHC) patients have very elevated serum miR-122 levels in the range of most patients with severe hepatic injury leading to acute liver failure [7]. In this research article, M. Sidorkiewicz et al. have enrolled 17 chronic hepatitis C patients with matching sera, PBMCs, and liver tissue specimen and found that liver (not PBMCs) miR-122 expression is positively correlated with HCV RNA load and IFN*γ* and reversely with LPL expression in CHC patients. Importantly, the results of the study firstly reveal the reverse correlation of miR-122 and LPL expression in liver. And the findings from the study would help us to have a deeper understanding of miR-122 in the occurrence and development of ACLF from CHC patients in future.

The research article titled “Serum Metabonomics Analysis of Liver Failure Treated by Nonbioartificial Liver Support Systems” aims to analyze the small molecular metabolic compounds of nonbioartificial liver for treatment of hepatic failure; and a total of 52 patients who meet the standard of artificial liver treatment for liver failure are enrolled. In this research article, significant changes in metabolic compounds of liver failure in the treatment before and after artificial liver are screened by using Ultra-Performance Liquid Chromatography-Mass Spectrometry (UPLC-MS), and related metabolic pathways are analyzed by MetaboAnalyst. Corresponding results suggest that artificial liver treatment could have significant effects on fatty acids and primary bile acid synthesis pathways; and the changes of fatty acid, primary bile acid synthesis pathway, and phenylalanine metabolic pathway are also significantly different among different artificial liver patterns of chronic liver failure. The findings from the study have somehow enriched our knowledge of the mechanism of Nonbioartificial Liver Support Systems in therapy of liver failure patients.

The research article titled “Transcriptome Analysis of Porcine PBMCs Reveals the Immune Cascade Response and Gene Ontology Terms Related to Cell Death and Fibrosis in the Progression of Liver Failure” aims to gain a deeper understanding of the transcriptional response of PBMCs following ALF. In this basic research article, Y. Zhang et al. have found that the dramatic PBMC transcriptome changes triggered by ALF progression are predominantly related to immune responses. And the enriched GO terms related to cell death, fibrosis, and so on, as indicated by PBMC transcriptome analysis, seem to be useful in elucidating potential key gene sets in the progression of ALF. As the authors have mentioned, a better understanding of these gene sets might be of preventive or therapeutic interest.

The clinical study titled “Good Tolerance of Citrate Accumulation due to Plasma Exchange among Patients with Acute-on-Chronic Liver Failure: A Prospective, Observational Study” is conducted among ACLF patients who plan to receive heparin anticoagulation during PE-centered therapy without filtration and dialysis. In this clinical research article, Y. Ma et al. have enrolled 54 patients, and they report that hypocalcemia (ionized calcium) and mild alkalosis are the main metabolic alterations. Additionally, no symptom associated with hypocalcemia occurred. To some extent, this study has suggested that citrate accumulation is well tolerated by ACLF patients who receive PE-centered therapy without filtration and dialysis. However, large sample size studies are required to confirm the present findings.

The research article titled “Alpha-Fetoprotein as a Predictive Marker for Patients with Hepatitis B-Related Acute-on-Chronic Liver Failure” aims to evaluate the prognostic effect of serum AFP on the prediction of HBV-ACLF outcomes. The results are basically consistent with previous similar studies. Recently, the role of stratified AFP is also concerned, and its power for predicting short-term survival of HBV-ACLF patients is superior to the absolute level of serum AFP [8]. For example, the Combining AFP quartiles with low serum sodium and high INR may better predict poor outcome in HBV-ACLF patients [8]. Previous reports have suggested that higher serum AFP levels could reflect a better regeneration ability of injured liver in patients [9, 10], and this may explain why high serum AFP level could predict a good outcome of ACLF patients.

The research article titled “Bacterial Infection and Predictors of Mortality in Patients with Autoimmune Liver Disease-Associated Acute-on-Chronic Liver Failure” aims to investigate bacterial infection and predictors of mortality among autoimmune liver disease-associated ACLF. In this retrospectively study, 53 ACLF patients and 53 patients without ACLF have been analyzed. Patients with bacterial infection have displayed a more severe condition than those without infection, and elevated CRP is found to be an accurate marker for diagnosing bacterial infection in autoimmune liver disease-associated ACLF patients. In fact, bacterial infection is an important precipitating factor of ACLF in one-third of ACLF, irrespective of the etiology of preexisting CLD [11, 12]. And the occurrence of bacterial infection is usually thought to be caused by systemic translocation of microbes from gut-derived organisms, impaired hepatic clearance mechanisms, and an ability of circulating immune cells to combat infectious cues [11, 13].
